# Factors targeting *MED12* to drive tumorigenesis?

**DOI:** 10.12688/f1000research.14227.2

**Published:** 2018-12-13

**Authors:** Jörn Bullerdiek, Birgit Rommel

**Affiliations:** 1 Institute of Medical Genetics, Medical Center, University of Rostock, Rostock, D-18057, Germany; 2 Human Genetics, University of Bremen, Bremen, D-28359 , Germany

**Keywords:** Mediator subcomplex 12 (MED12), mutations, uterine leiomyomas, fibroadenomas of the breast, chronic lymphocytic leukemia (CLL), multiple tumors, DNA-RNA hybrids, Staphylococcus aureus

## Abstract

Mediator Complex Subunit 12 (MED12) is part of the transcriptional preinitiation machinery. Mutations of its gene predominantly occur in two types of highly frequent benign tumors, uterine leiomyomas and fibroadenomas of the breast, where they apparently act as driver mutations. Nevertheless, their presence is not restricted to benign tumors having been found at considerable frequencies in uterine leiomyosarcomas, malignant phyllodes tumors, and chronic lymphocytic leukemia also. Most of the mutations are located within exon 2 of the gene but in rare cases the intron 1/exon 2 boundary or exon 1 are affected. As to their type, predominantly single nucleotide exchanges with a hotspot in one codon are found, but small deletions clustering around that hotspot also are not uncommon. These latter deletions are leaving the open reading frame intact. As to the types of mutations, so far no apparent differences between the tumor entities affected have emerged. Interestingly, this pattern with small deletions clustered around the hotspot of single nucleotide exchanges resembles that seen as a result of targeted gene editing. In contrast to other driver mutations the percentage of *MED12*-mutation positive tumors of independent clonal origin increases with the number of tumors per patient suggesting unknown etiological factors supporting site specific mutagenesis.  These factors may act by inducing simultaneous site-specific double strand breaks the erroneous repair of which may lead to corresponding mutations. As inducers of DNA damage and its repair such as foreign nucleic acids of the microbiome displaying sequence homology to the putative target site might play a role. Interestingly, a 16 base pair homology of the hotspot to a putative terminator base-paired hairpin sequence of a Staphylococcus aureus tRNA gene cluster has been noted which might form R-loop like structures with its target sequence thus inducing said changes.

## Introduction

Surprisingly, the most common mutation in human tumors does not affect one of the famous suspects in the field (for review see ( Vogelstein *et al.*, 2013)) but the much less well-known gene encoding Mediator Complex Subunit 12 ( *MED12*). MED12 is part of the transcriptional preinitiation complex CDK8 ( Elmlund *et al.*, 2006) and encoded by a gene that maps to the X-chromosome at Xq13.1. One obvious reason why its mutations so far have gained much less interest than those of other genes frequently mutated in human tumors is that they affect, to a large extent, benign tumors. Moreover, within malignant tumors, they are virtually absent from most of the predominant epithelial neoplasms like cancers of colon, breast, and lung ( Kandoth *et al.*, 2013) whereas they have been found at considerable frequencies in some non-epithelial malignant tumors having a possible origin from benign precursor lesions.

As to the benign tumors, however, mutations of *MED12* occur as apparent driver mutations in a predominant subset of human uterine leiomyomas ( Mäkinen *et al.*, 2011; Markowski *et al.*, 2012; McGuire *et al.*, 2012), constituting the by far most frequent human symptomatic tumors of all. Likewise, these mutations are also found in a large subset of fibroadenomas of the breast ( Lim *et al.*, 2014; Piscuoglio *et al.*, 2015; Yoshida *et al.*, 2015), another frequent benign tumor which occurs predominantly in young and middle-aged women. Interestingly, they are not restricted to benign tumors but also frequently seen in their malignant counterparts, i.e. uterine leiomyosarcomas ( Kämpjärvi *et al.*, 2012; Markowski *et al.*, 2013a; Pérot *et al.*, 2012) and malignant phyllodes tumors ( Nagasawa *et al.*, 2015; Piscuoglio *et al.*, 2015; Yoshida *et al.*, 2015). Also, their presence in malignant tumors suggests that, albeit as a very rare event, certain additional mutations can trigger malignant transformation within formerly benign tumors harboring *MED12* mutations. Apart from solid tumors, *MED12* mutations recently were also detected in a significant percentage of roughly 5–9% of chronic lymphocytic leukemias (CLL) ( Guièze *et al.*, 2015; Kämpjärvi *et al.*, 2015; Wu *et al.*, 2017a). Furthermore, the same type of *MED12* mutation was found in two canine vaginal leiomyomas ( Markowski *et al.*, 2013a).

A closer look at the *MED12* mutations does not reveal apparent differences between the type of mutations when comparing the different tumor entities. While for CLL only a few cases have been reported so far, the mutations in uterine leiomyomas and fibrodadenomas of the breast predominantly are clustered in the 5´ region of exon 2 of the gene with only a few mutations affecting the intron 1-exon 2 boundary or, more rarely, exon 1 or the exon 1-intron 1 boundary. Most of them are single base exchanges clearly clustered at two nucleotides of codon 44 where, albeit with different frequencies, guanins are found to be replaced by either A, C, or T. Besides these single base replacements, deletions and, more rarely, indels, usually affecting exon 2 or the intron 1/exon 2 boundary are found which always leave the reading frame intact indicating that the mutations do not exert their tumor driving potential simply by abrogating the function of MED12. *MED12* maps to the X-chromosome and, as revealed by cDNA sequencing, the mutations are apparently restricted to the active X-chromosome ( Mäkinen *et al.*, 2011; Markowski *et al.*, 2012; McGuire *et al.*, 2012).

We feel that for several reasons this highly frequent type of mutation might point to an unusual mechanism of mutagenesis underlying the development of the corresponding tumors. These reasons will be discussed herein and a hypothesis based on target-specific mutagenesis will be presented. Starting with a short introduction of the main tumors affected by *MED12* mutations we will then address the molecular pathogenesis of uterine leiomyomas along with its clinical correlations followed by an in depth analysis of the pattern of *MED12* mutations. Finally, we will present a hypothesis why these data indicate a so far unknown etiological mechanism favoring these particular highly frequent somatic alterations of the genome.

## Introducing three tumor entities displaying *MED12* mutations

Besides a few very rare tumors, three main tumor entities are often affected by mutations of *MED12*. First, these three entities, i.e. uterine leiomyomas (fibroids), fibroadenomas of the breast, and chronic lymphocytic leukemias, will be introduced.

### Uterine leiomyomas - the most frequent symptomatic human tumors

**Figure 1.**
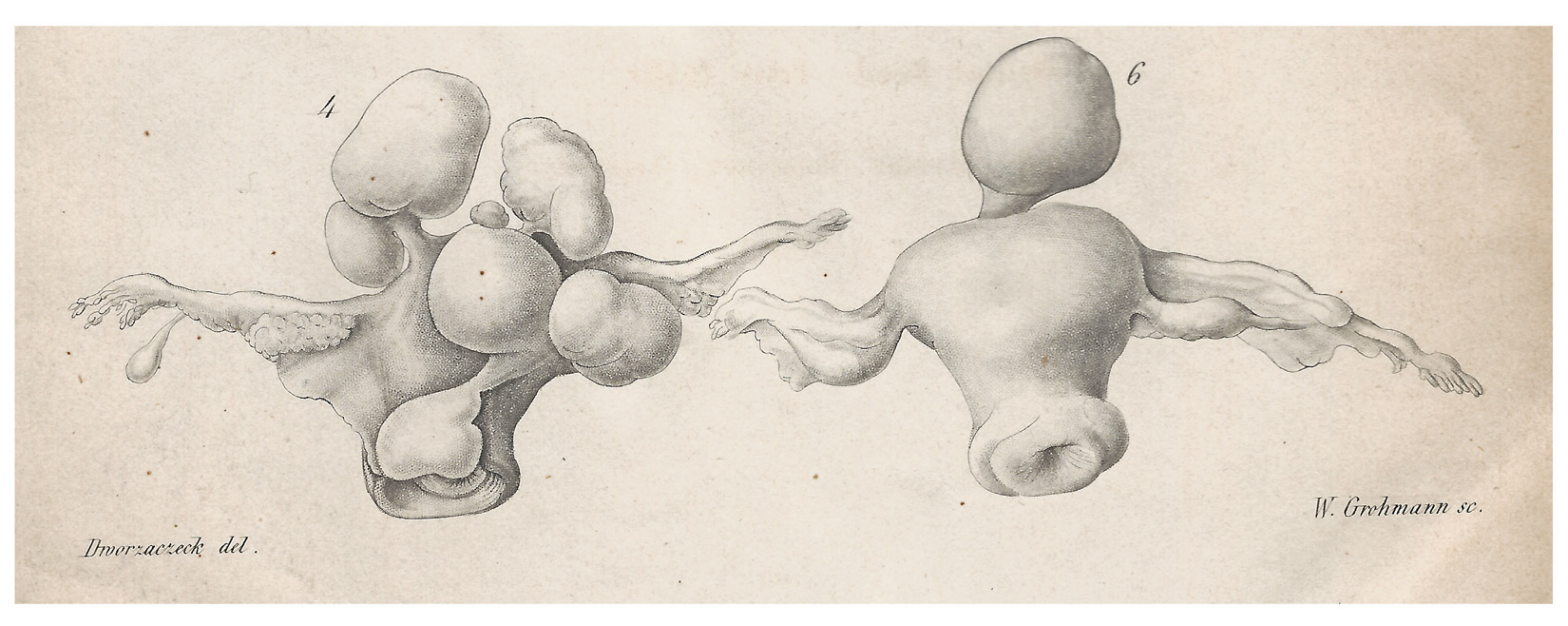
Uterine leiomyomas in the scientific literature of the 19th century. Illustration of a uterus carrying multiple leiomyomas of various sizes and location (4, left) and uterus with a large pedunculated subserous leiomyoma (right, 6). Copperplate engraving from Virchow´s series of lectures entitled “Die krankhaften Geschwülste” (“Morbid neoplasms”)(Hirschwald, Berlin, 1864/65).

Uterine leiomyomas (UL) are benign smooth muscle tumors of myometrial origin with an apparently very low tendency to undergo malignant transformation. Depending on the location, the difference between submucosal, intramural, and subserosal UL can be distinguished. Roughly 40–70% of women in their reproductive age will develop UL with a well-documented higher prevalence among women of African and African-American origin. In this group, the UL develop on average at younger ages ( Laughlin *et al.*, 2010). In general, epidemiological studies have a revealed a lower risk of fibroid development associated with parity which may be related to tissue remodelling during pregancy ( Baird & Dunson, 2003). Leiomyomas are often of large size and, as a matter of debate for more than 100 years, ( Figure 1), multiple nodules occur at almost the same frequency or even more frequently than single nodules.

Nevertheless, the majority of patients with UL are without symptom. The remaining 20–30% of the patients suffer from symptoms like in particular heavy menstrual bleeding, increased menstrual periods, reduced fertility, and pelvic pressure and pain. During menopause, UL cease growing and even shrink. Nevertheless, despite their benign nature UL are the most symptomatic human tumors of all and repeatedly have been reported even in mummies with the oldest of them dating back to the Middle Neolithic age (3,200–2,500 BC) ( Fornaciari & Giuffra, 2012). Surgery (hysterectomy or myomectomy) is a common type of treatment, but a variety of other non-surgical methodologies to treat UL are available (for review see: ( Williams, 2017)).

While the etiology of these frequent tumors remains unclear ( McWilliams & Chennathukuzhi, 2017), a bit more is known about pathogenetic factors. As to basic principles of their molecular pathogenesis, UL behave like the vast majority of other benign and malignant tumors. This includes a monoclonal origin which is triggered by so-called driver mutations. In the case of multiple UL almost every single nodule has been found to be of independent clonal origin ( Mashal *et al.*, 1994). Accordingly, different nodules are usually characterized by different driver mutations in these cases. The role of myometrial stem cells as targets of driver mutations has been reviewed by Commandeur *et al*. ( Commandeur *et al*., 2015).

Moreover, genetic subtypes, apparently belonging to different groups of driver mutations, exist. While *MED12* mutations, as a group, constitute the most predominant genetic subtype ( Mäkinen *et al.*, 2011), another frequent subgroup of uterine leiomyomas carries rearrangements of the gene encoding the architectural transcription factor High Mobility AT-hook 2 ( *HMGA2*), as a rule reflected by cytogenetically visible chromosomal translocations ( Schoenmakers *et al.*, 1995). Of note, the driver mutations of both subgroups occur in a mutually exclusive manner ( Markowski *et al.*, 2012). Besides these subgroups, other, more rare but also independent genetic subgroups of UL such as one characterized by either germline (hereditary leiomyomatosis and renal cell cancer (HLRCC), OMIM 605839) ( Bayley *et al.*, 2008) or somatic loss-of-function mutations of *Fumarate Dehydrogenase* ( *FH*) seem to exist ( Kämpjärvi *et al.*, 2016).

### Fibroadenomas of the breast - Frequent benign tumors in adolescent and young women

Fibroadenomas of the breast are common benign tumors histologically composed of both stromal and epithelial components preferentially occurring in adolescent and young women. Their general incidence may be in the range of 10% in the corresponding age groups. Multiple tumors are not rare with some 10–15% of the patients having more than one FA.

Their name fibroadenomas (FA) and their classification as fibroepithelial tumors suggest a biphasic nature of these neoplasms. Nevertheless, as to their pathogenesis this classification seems to be misleading at least in the majority of cases because mutations are restricted to the stromal component of the tumors ( Mishima *et al.*, 2015). Between 50% and 60% of the FA harbor *MED12* mutations ( Lim *et al.*, 2014; Mishima *et al.*, 2015; Pfarr *et al.*, 2015) Histologically, the occurrence of *MED12* mutations correlates highly significant with the so-called intracanalicular growth pattern ( Mishima *et al.*, 2015; Pfarr *et al.*, 2015). Interestingly, *MED12* mutations have also been found in considerable percentages of other breast tumors of presumed stromal origin Phyllodes tumors and malignant Phyllodes tumors. In these tumors, the types of *MED12* mutations are not obviously discernible from those observed in UL and FA.

### Chronic lymphocytic leukemias - most frequent leukemia in adults

In Western countries, chronic lymphocytic leukemia (CLL) is the most common type of leukemia in adults. The American Cancer Society expects an estimated number of about 20,940 new cases of CLL in the United States in 2018 with about 4,510 CLL-related deaths. Overall, CLL accounts for about one-quarter of the new cases of leukemia. The average person's lifetime risk of getting CLL is about one in 175 (0.57%). The average age at the time of diagnosis is around 70 years, with men slightly more often affected than women. Frequently, the disease is detected in patients not yet showing any severe symptoms and in its early stages patients often undergo a ‘watch and wait’ period prior to starting therapy.

As to its pathogenesis CLL is a monoclonal leukemia of B-cell origin with a number of subsets that can be distinguished based on their genetic characterization, apparently pointing to different driver mutations. The genetic alterations also allow the stratification of groups which may require different therapeutic approaches. As a valid predictive parameter, deletions and mutations of the *TP53* gene are associated with a worse prognosis than other types of genetic changes and influence therapeutic decisions. Recently, mutations of *MED12* have been added to the list of potential driver mutations in CLL. Across the sub-types, they constitute a relatively rare genetic group affecting roughly 5–9% of CLL patients ( Guièze *et al.*, 2015; Kämpjärvi *et al.*, 2015; Wu *et al.*, 2017a). Kämpjärvi *et al*. ( Kämpjärvi *et al.*, 2015) have presented evidence that *MED12* mutations may represent a marker of worse prognosis.

## A closer look at the molecular pathogenesis of uterine leiomyomas

According to the high prevalence of uterine leiomyomas *MED12* mutations are by far best investigated in this tumor type. Thus, we have now characterized the subset UL affected and described how they can be distinguished from other types of UL.

### Leiomyomas with *MED12* mutation constitute their own genetic subtype which is also characterized by a distinct clinical and histopathological appearance

There is ample evidence that *HMGA2* rearrangements and *MED12* mutations occur mutually exclusively in UL and thus constitute independent driver mutations ( Markowski *et al.*, 2012). Similarly, somatic *MED12* mutations and biallelic *Fumarate Hydratase* ( *FH*) inactivation occur in mutually exclusive manner in both HLRCC syndrome-associated and sporadic uterine leiomyomas suggesting that the latter constitutes a third small group with an independent molecular pathogenesis ( Kämpjärvi *et al.*, 2016). Accordingly, each of these genetic alterations alone as a driver mutation seems to be sufficient to induce the development of an UL without requiring any further mutations.

As to rearrangements of *HMGA2* and mutations of *MED12*, the transcriptome of tumors of both groups clearly differs with *MED12* mutation–positive and *HMGA2*-overexpressing samples clustering in distinct branches ( Mehine *et al.*, 2013). Accordingly, both mutations allow the two major genetic subtypes of UL to be distinguished, and the question arises whether or not the genetic subtypes are also reflected by a different clinical behavior and histopathology.

**Figure 2.**
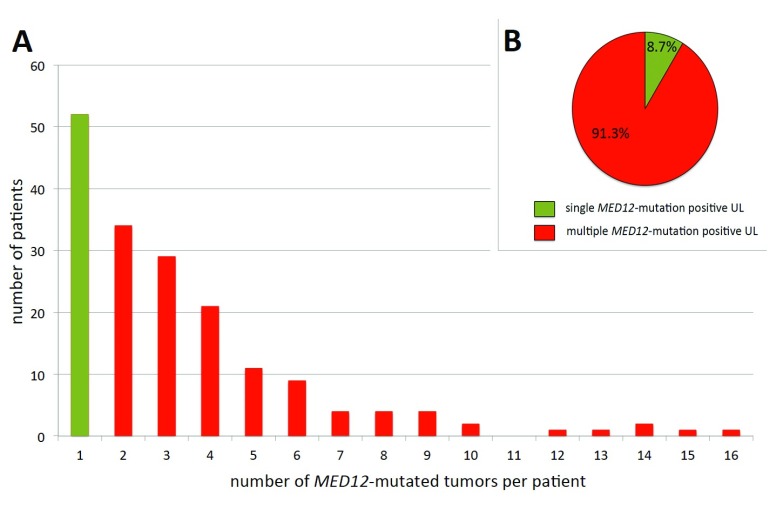
Solitary and multiple *MED12*-mutation-positive uterine leiomyomas. Abscissa: number of UL/patient, ordinate: number of patients in the corresponding category. For this diagram data on *MED12* alterations published by Heinonen *et al*. ( Heinonen *et al.*, 2017) have been used. Overall, 52/176 women carried a single *MED12*-mutated UL compared to 124/176 with more than one such tumor ( **A**) and proportion of *MED12*-mutated UL appearing as single tumor (52/599) *vs*. those accompanied by at least one other *MED12*-mutated UL (547/599) ( **B**). Data according to Heinonen *et al*. (2017).

Tumors carrying *HMGA2* rearrangements are usually solitary and, on average, of larger size than those with *MED12* mutations, also usually presenting as single tumors ( Markowski *et al.*, 2014a) whereas the latter are smaller and often co-occur with other clonally independent nodules of the same genetic type ( Mäkinen *et al.*, 2011; Markowski *et al.*, 2012; Markowski *et al.*, 2014a). Among the women affected by *MED12*-mutation positive UL, more than two-thirds had more than one nodule ( Heinonen *et al.*, 2017) ( Figure 2A) of this type. Even more impressive, in the same study 52 (8.7%) *MED12*-mutation positive tumors made their appearance as the sole tumor with this mutation, while 547 (91.3%) tumors were associated with at least one other tumor of this genetic subgroup ( Figure 2B).

To explain the high frequency of *MED12* mutated tumors among multiple leiomyomas, Heinonen *et al*. speculated that "the multiplicity of *MED12*-mutation-positive leiomyomas may derive from genetic predisposition and/or environmental factors rendering the myometrium susceptible to selection for *MED12* mutations" ( Heinonen *et al.*, 2017). However, as outlined later herein, the association of multiple tumors with *MED12* mutations may be a key to the etiology of this type of UL.

Furthermore, *MED12*-mutated UL are also significantly associated with a subserous location compared to UL lacking this mutation ( Heinonen *et al.*, 2017). As to histopathological features, a recent study by Wu *et al*. revealed that approximately 90% of the cells in *HMGA2*-rearranged UL were smooth muscle cells showing an overexpression of the protein, while in *MED12*-mutated UL a similar number of smooth muscle cells and other cells, i.e. mostly tumor-associated fibroblasts, were detected. These latter fibroblasts were lacking *MED12*-mutations ( Wu *et al.*, 2017b) and thus apparently can be classified as by-stander cells. This fits with an earlier observation that in cell cultures of leiomyomas with *MED12*-mutations a rapid disappearance of mutated cells was seen that became replaced by wild-type cells, thus challenging the results of a variety of *in vitro* experiments on the biology of UL ( Bloch *et al.*, 2017; Markowski *et al.*, 2014b).

Besides UL, the occurrence of *MED12* mutations has been well-documented in malignant uterine smooth muscle tumors (leiomyosarcomas) and smooth muscle tumors of uncertain malignant potential (STUMP), too ( Holzmann *et al.*, 2015; Pérot *et al.*, 2012). Hence, an origin of these tumors from pre-existing UL has been suggested. In contrast, similar cases with *HMGA2* rearrangements have not been reported yet.

### The percentage of *MED12*-mutated tumors is positively correlated with the total number of tumors per patient

**Figure 3.**
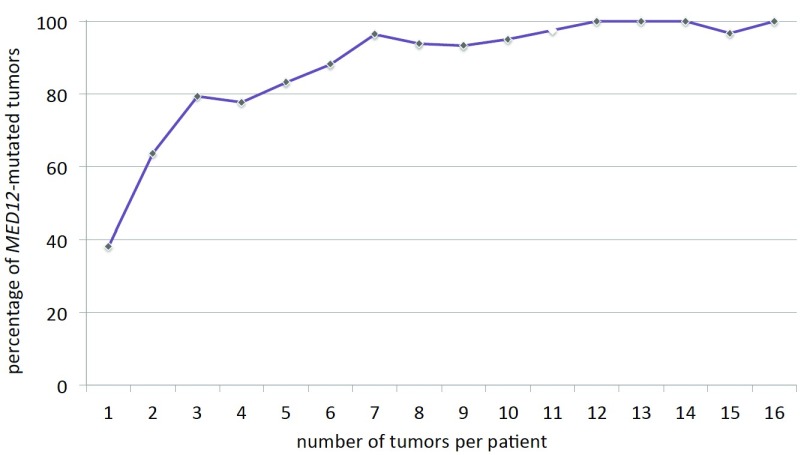
Number of uterine leiomyomas per patient and percentage of *MED12*-mutation positive tumors.

To gain further insight into the biology of *MED12*-mutated UL, Heinonen *et al*. have undertaken a systematic attempt to check all feasible distinct tumors with a size of 1 cm or larger in diameter from hysterectomy uteri for *MED12* mutations ( Heinonen *et al.*, 2017). In their study, 599 out of 763 leiomyomas carried *MED12* mutations (79%). Next, the data provided by the study of Heinonen *et al*. ( Heinonen *et al.*, 2017) have been used to analyze the number of *MED12*-mutation positive UL per patient. While it was shown before that in the majority of patients having surgery *MED12*-mutated tumors do not make their appearance as single nodules but instead are accompanied by other yet clonally independent tumors of this same genetic type (cf. Figure 2), we were also interested to see how the number of tumors per patient is distributed in this genetically distinct group of tumors. Obviously, a slow decrease of the number of *MED12*-mutation positive tumors is noted ( Figure 2A). Nevertheless, from these figures it is not possible to draw conclusions on the overall frequency and distribution of *MED12*-positive UL in the population because the results are biased by their restriction to symptomatic patients who had undergone surgical treatment. Nevertheless, they correspond more or less to the tumor numbers in general as seen from numerous other studies and thus the question arises if the *MED12*-positive tumors can be distinguished from the remaining UL as suggested by previous estimations (cf. Figure 2). Thus, we have next investigated if, in the case of multiple tumors, the percentage of *MED12*-mutation positive UL remains constant independent of the total number of tumors per patient. Surprisingly, it was noted that with a growing number of tumors, the percentage of *MED12*-mutated tumors clearly increased. Among solitary leiomyomas, only less than 40% of the tumors carried *MED12* mutations but their frequency was approaching nearly 100% if twelve or more tumors were present per patient ( Figure 3). Thus, in contrast to other mutations, those of *MED12* seem to become more likely with an increase of the number of tumors.

Increasing percentage of *MED12*-mutation positive uterine leiomyomas (ordinate) with the number of tumors per patient (abscissa). Open rhombus indicates an interpolated value because no patients with 11 UL were present. For this diagram data on *MED12* alterations published by Heinonen *et al*. (2017) have been used.

Along with previous data this distribution confirms that the occurrence of multiple leiomyomas nearby can be exclusively attributed to just one genetic mechanism, i.e. *MED12* mutations. For example, in a study by Markowski *et al*. only 26/179 (14.5%) of *MED12*-mutated UL were single tumors while the corresponding number for *HMGA2*-rearranged UL was 14/20 (70%). In none of the latter cases, a *HMGA2*-rearranged UL was accompanied by another one with *HMGA2* rearrangement ( Markowski *et al.*, 2014a). This contradicts a statement by Mehine *et al*. that a shared clonal origin as a common feature of leiomyomas not carrying *MED12* mutations offers one explanation for the common occurrence of multiple concurrent lesions ( Mehine *et al.*, 2015). Instead, a multitude of fibroids mainly appears to be a problem almost exclusively restricted to *MED12*-mutation positive tumors and we thus decided to analyze and compare the different *MED12* mutations in more detail.

## A closer look at the patterns of *MED12* mutations seen in various benign and malignant tumors

*MED12* mutations occur in uterine smooth muscle tumors, fibroepithelial tumors of the breast and in chronic lymphocytic leukemia. As a rule, only subsets display these genetic abnormalities which range from the predominant alterations in UL to those detected only in a small percentage of cases in CLL. So far, there is no evidence that the tumors affected by *MED12* mutations in the three tumor entities described above differ with respect to their types of mutations. It has been speculated that unknown factors favor the occurrence of these particular mutations. To get further ideas on factors favoring them, the patterns of mutations have now been analyzed in more detail and compared between the different tumor entities paying particular attention to the small deletions occurring in exon 2 or at the intron 1-exon 2 boundary.

**Figure 4.**
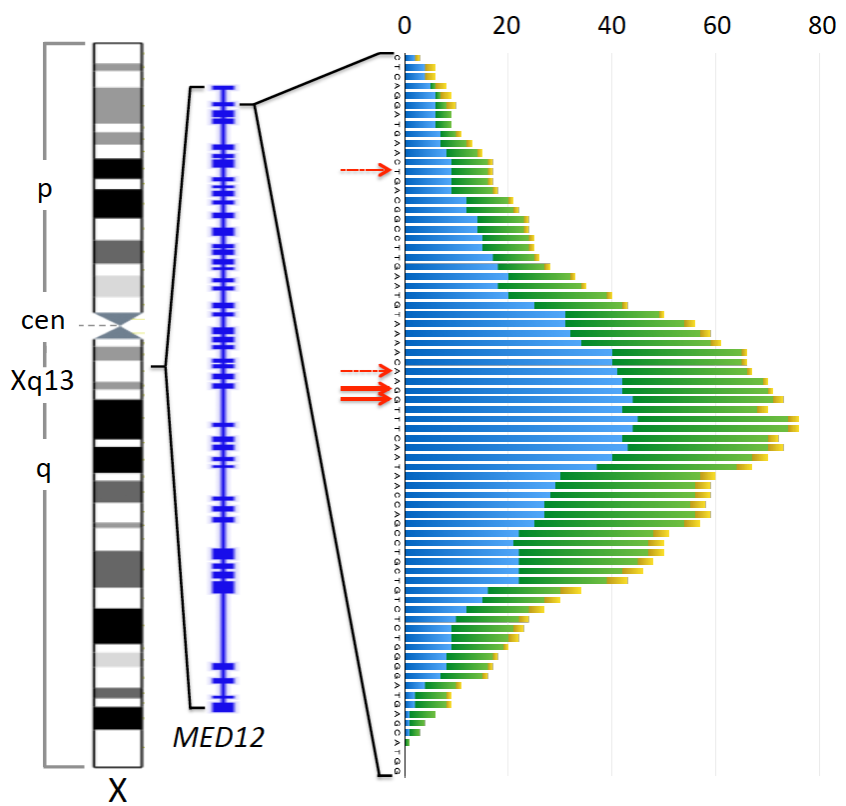
Patterns of *MED12* deletions in uterine leiomyomas, fibroepithelial breast tumors, and chronic lymphocytic leukemia. Left to right: Ideogram of the X-chromosome (commons.wikimedia.org), exon-intron structure of *MED12* (NCBI map viewer), and plot depicting frequency of deletions at each position around the preferred site of single nucleotide exchanges (red solid arrows) seen in uterine smooth muscle tumors (blue), fibroepithelial tumors of the breast (green), and chronic lymphocytic leukemia (yellow). Deletions are plotted across all deleted base positions. Minor preferred sites of single nucleotid exchanges within exon 2 are indicated by dashed red arrows. For this diagram data on *MED12* deletions from the following articles have been used: ( Guièze *et al.*, 2015; Kämpjärvi *et al.*, 2015; Lim *et al.*, 2014; Markowski *et al.*, 2013b; Mishima *et al.*, 2015; Nagasawa *et al.*, 2015; Ng *et al.*, 2015; Pfarr *et al.*, 2015; Yoshida *et al.*, 2015) only those deletions beginning and ending in the displayed region have been considered.

In most cases single nucleotide exchanges are found with a clear predominance of those affecting nucleotides 130 and 131 belonging to codon 44. Less frequently other codons are mutated. Besides single nucleotide exchanges, deletions of small segments of the gene with varying sizes as well as indels affecting exon 2 or the intron1-exon 2 boundary are seen in some cases. As a rule, however, the transcript though affected by the deletions remains in frame. As to these latter genomic alterations accounting for roughly 15% of *MED12* mutations, we have analyzed the positions of the deleted bases from a variety of papers analyzing UL, fibroepithelial tumors, and CLL. Adding the number of deleted bases per each position reveals an almost symmetric distribution that is clustered around the hotspot of single nucleotide exchanges ( Figure 4). Accordingly, e.g. in the Heinonen *et al*. series, deletions encompassing nucleotides 129–134 or part of them are clearly more frequent than those outside this fragment. This clustering becomes evident when analyzing uterine smooth muscle tumors and fibroepithelial tumors of the breast alone, whereas in case of CLL only a few cases have been reported in the literature so far. In a previous study by our group, the beginning of the *MED12* deletions observed in uterine smooth muscle tumors was mostly located within exon 2 but in rare cases also upstream of the splice site within intron 1. Their size mainly ranged between 3 and 36 bp with a clear predominance of 15 and 18 bp ( Markowski *et al.*, 2013b). Of note, an analysis of the data provided by Heinonen *et al*. for UL revealed that as very rare exceptions even larger deletions as well as those residing in exon 1 can occur.

As to size and position of these deletions, there is also no obvious difference between UL, fibroepithelial tumors of the breast, and CLL ( Figure 4). Overall, this pattern of small deletions of various sizes clustered around the hotspot of single nucleotide exchanges in general could be explained by bias caused by a higher proliferative activity of UL characterized by deletions encompassing the central hotspot area which accordingly would be of larger size and more likely to become symptomatic. Nevertheless, according to our evaluation of the Heinonen series Heinonen *et al*. (2017), the average size of UL with deletions encompassing any of the nucleotides 129–134 (average: 3.32) does not significantly differ from those having deletions outside this region (average: 3.35). Thus, such an obvious explanation seems less likely to explain the distribution which, however, resembles the results of genome editing based on targeted double-stranded breaks as for example those resulting from the usage of the CRISPR/Cas9 system (e.g. cf. Paquet *et al.*, 2016).

**Figure 5.**
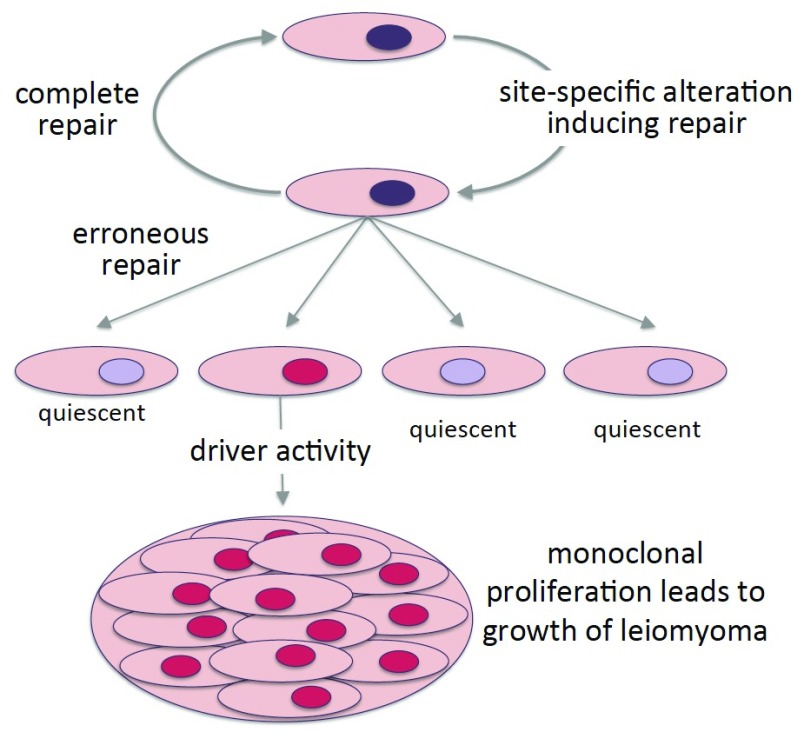
*MED12* mutations in uterine smooth muscle tissue. Model illustrating the occurrence and selection of *MED12* mutations during the course of leiomyoma development. The scheme suggests that of a larger number of *MED12*-mutations only those associated with a gain of function act as driver mutations giving rise to UL.

If these mutations indeed arise by certain types of repair of site-specific DNA changes, one might expect that many other mutations occur in the target region of *MED12*. Of these, only the “active ones”, i.e. those leaving the open reading frame intact and driving tumorigenesis, will lead to a clonal proliferation of their target cell giving rise to an UL whereas cells with other mutations of the hotspot region will remain quiescent or even become apoptotic ( Figure 5). Therefore, future studies aimed at the detection of these “non-driving” mutations in single cells, especially from patients suffering from a multitude of UL, may be a reasonable attempt. However, from the pattern of nucleotide exchanges and deletions, a commonly affected sequence can be depicted that may be related to the etiology of UL.

## Hypothesis and opinion

*MED12* mutations constitute highly frequent driver mutations in uterine leiomyomas and fibroadenomas, i.e. two tumor entities that occur almost exclusively in middle-aged and young women, respectively. In uterine leiomyomas, they even represent the by far most frequent genetic subtype with a clearly preferential occurrence in the case of multiple tumors. This is in sharp contrast to the other main genetic subtype of UL characterized by rearrangements of *HMGA2* usually making its appearance in solitary nodules not accompanied by other tumors of the same genetic subtype.

**Figure 6.**
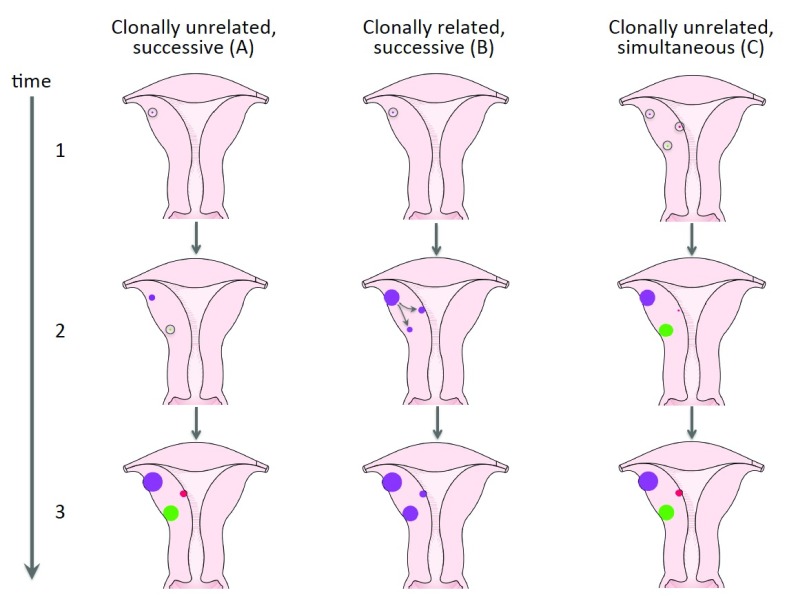
Different models of time and clonality of uterine leiomyoma development. Three alternative models explaining the development of multiple uterine leiomyomas are depicted: ( **A**) corresponds to a *prima facie* model of myomagenesis with multiple independently developing nodules having a different growth rate. ( **B**) Illustrates the development of multiple clonally related tumors from one common predecessor as described by Mehine *et al*. ( Mehine *et al.*, 2015) for *MED12*-mutation negative UL. ( **C**) offers an alternative explanation for the growth of multiple clonally unrelated nodules with simultaneous initiation but a different growth rate. Open circles indicate single mutated cells of origin affected by driver mutation.

It seems difficult to explain these findings just by independent random mutations followed by their selection. Nevertheless, additional factors favoring this multitude of tumors with independent clonal origin carrying the same type of mutation have remained enigmatic. After myomectomy, such factors may also account for the risk of recurrences that clearly increases with the number of UL that had been removed ( Doridot *et al.*, 2001; Fauconnier *et al.*, 2000). Heinonen *et al*. (2017) have speculated that either genetic predisposition or environmental factors rendering the myometrium susceptible to selection for *MED12* mutations may contribute to the multiplicity of *MED12*-mutation positive tumors. To describe the development of multiple tumors, those two explanations are well-compatible with a model of clonally unrelated nodules that occur successively and are endowed with a different growth rate as depicted in Figure 6A. Another alternative explaining the multiplicity of UL is the occurrence of clonally related nodules with marked genetic evolution as shown by Mehine *et al*. ( Mehine *et al.*, 2015) for UL lacking *MED12* mutations and depicted here as Figure 6B. Nevertheless, a variety of studies indicate that most tumors with *MED12* mutations are not clonally related and the multiplicity of these lesions thus needs other explanations. In addition to these models, the potential roles of infectious agents warrant consideration. The infection may lead to a synchronous initiation of multiple clonally independent lesions endowed with a different growth potential ( Figure 6C).

As possible factors certain types of bacteria, e.g. those involved in reproductive tract infections (RTIs), have long been suggested to be a cause of UL (e.g. ( Witherspoon & Butler, 1934)). In the United States both reportable RTIs (i.e. chlamydia and gonorrhea) and fibroids disproportionately burden African American women which lead to the conclusion that the growth of fibroids might be triggered by inflammatory infections associated with the RTIs. Nevertheless, when exploring the relationship between self-reported RTIs and fibroid size, number, and total volume Moore *et al*. did not find strong associations ( Moore *et al.*, 2015). In a recent contribution by the same group women seropositive for genital *Chlamydia trachomatis* were even found to be less likely to have fibroids ( Moore *et al.*, 2018). In line with these findings, in the study by Heinonen *et al*. neither a history of pelvic inflammatory disease (PID) nor of *Chlamydia* infection was found to be significantly associated with the *MED12*-type UL while PID turned out to be significantly associated with the occurrence of *MED12*-wild type UL (p 0.0024) ( Heinonen *et al.*, 2017).

Akin to bacteria, viruses have also been suggested to be involved in the development of UL ( Bullerdiek, 1999). For example, *EBV* is known as a factor associated with the development of extra uterine smooth muscle tumors in HIV and post-transplant patients (see e.g. ( Miettinen, 2014; Purgina *et al.*, 2011; Ramdial *et al.*, 2011)). However, so far no association between *EBV* and uterine leiomyomas has been demonstrated. As to another virus of the Herpes group, a recent study failed to reveal a significant association between *HSV-2* seropositivity and the presence of fibroids ( Moore *et al.*, 2016) and in general no convincing evidence for involvement of viruses in the pathogenesis of UL has been presented.

While infectious diseases as etiological agents of UL repeatedly have been assumed as such the question arises as to how they could act by site-specific targeting the hotspot region of mutations residing within exon 2 of *MED12* and which infectious agents are possible candidates. We will herein present the opinion that the interaction between the human DNA and foreign nucleic acids derived from the infection plays a causal role. As an unorthodox hypothesis stimulating further discussions, we would like to advance the hypothesis that the *MED12* mutations result from cleavage of R-loop structures. By definition, R-loops are derived from double stranded DNA where one strand forms a stable DNA-RNA hybrid helix whereas the former associated DNA strand remains single-stranded. R-loops with an “exposed” stretch of single-stranded DNA can give rise to instability and DNA double-strand breaks ( Aguilera & Gómez-González, 2017; Freudenreich, 2018; Su & Freudenreich, 2017). While the hybrid helix is usually composed of DNA with endogenous RNAs it seems possible that such helices can be formed with foreign RNA as well. To this end, it has been hypothesized that circulating exogenous RNA sequences after their uptake may influence the function of cells through miRNA-like mechanisms ( Wang *et al.*, 2012) suggesting direct influences of these sequences of the microbiome on its host´s cells.

**Figure 7.**
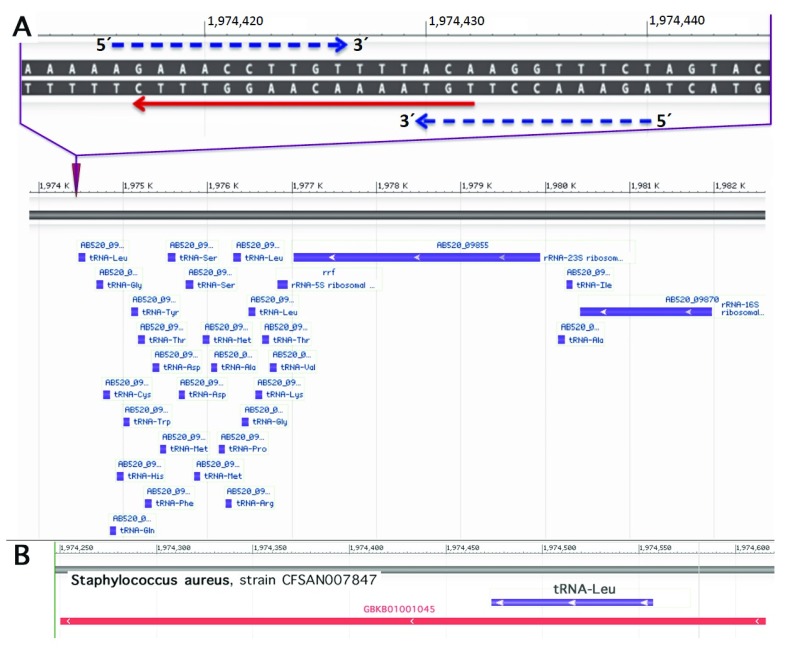
Sequence adjacent to a 27-tRNA gene cluster of *Staphylococcus aureus* similar to the *MED12* hotspot and a corresponding transcript. Upper part: Homology of the human *MED12* hotspot region (red arrow) with the sense strand of the sequence of the *Staphylococcus aureus* 27-tRNA gene cluster immediately 3’ to a rRNA operon ( Green & Vold, 1993). Blue dashed arrows indicate a palindromic sequence. Sequence from *Staphylococcus aureus* strain CFSAN007847 chromosome, complete genome; GenBank: CP017684.1; GenBank: FASTA; NCBI Blast. Lower part: bacterial tRNA and rRNA genes of the operon adjacent to the site of homology are shown in blue ( **A**). Transcript containing parts of the of the operon and its 3´ vicinity including the homologous region ( Tan *et al*., 2015: GeneBank accession GBKB01001045)( **B**).

**Figure 8.**
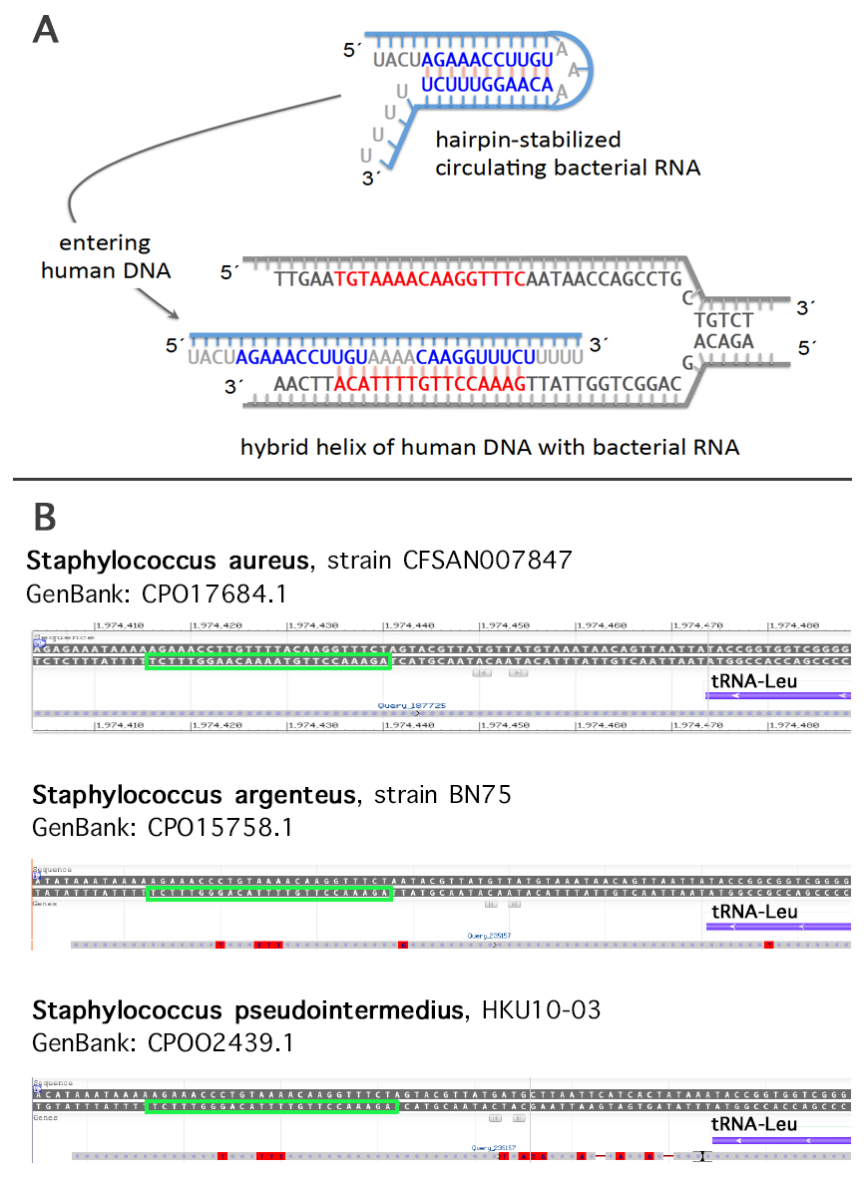
Model depicting the interaction between bacterial RNA with the *MED12* mutational hotspot and similarity of the corresponding palindromic structure in different species of *Staphylococcus*. As an example for the putative sequence of the human microbiome inducing site specific mutations this scheme refers to the sequence depicted in Figure 7 ( **A**). A similar palindromic structure is present at the same position of some other *Staphylococcus* species as shown here as examples for *S. argenteus* and *S. pseudointermedius*, respectively (green squares). ( **B**)

To search for possible sequence homologies we have depicted a target region 5´TGTAAAACAA **GG**TTTCAATAAC3´ covering 10 nucleotides upstream and downstream each of the two c. 130 and c. 131 (GG), respectively (cf. Figure 5). Of the resulting list with at least 15 identical nucleotides, a variety of human pathogens have been identified as e.g. *Bacteroides fragilis* (nt 2-20), *Klebsiella pneumoniae* (nt 2-20), *Escherichia coli* (nt 3-20), *Vibrio vulnificus* (nt 6-22), *Staphylococcus aureus* (nt 6-22, and nt 1-16, respectively), *Staphylococcus argenteus* (nt 6-22), and *Clostridium botulinum* (nt 3-19). When searching for abundantly expressed sequences an interesting candidate emerged. A 16-base pair sequence identical to the sense strand of the sequence of a *Staphylococcus aureus* 27-tRNA gene cluster immediately 3’ to an rRNA operon ( Green & Vold, 1993) was noted ( Figure 7A). A transcript containing this sequence has been described ( Tan *et al*., 2015, GeneBank accession GBKB01001045, Figure 7B). The homology covers a palindromic sequence which may act as a terminator of transcription ( Green & Vold, 1993) and may also lead to the formation of a hairpin structure stabilizing the RNA molecule ( Figure 8A). Of note, very similar palindromic sequences have been found at the same position of the genomes of other species of *Staphylococcus* ( Figure 8B). Nevertheless, whereas the palindromic arms have high similarity the central part, the three AAA have undergone transversion to TTT. Are these molecules likely to exist as circulating RNAs? *Staphylococcus aureus* belongs to the phylum of *Firmicutes* in general constituting the third most abundant sequence population in human plasma with a significant number of the reads mapping to various bacterial ribosomal RNAs and tRNAs ( Wang *et al.*, 2012). More specifically, evidence has been presented that RNA species from *Staphylococcus* are commonly present in blood ( Leung & Wu, 2015). Of note, *Firmicutes* are not rarely found to be part of the microbiota of the female genital tract with *Staphylococcus* sp. e.g. being the most abundant genus recovered from the fallopian tubes ( Pelzer *et al*., 2018). This presence is not necessarily supposed to result from an ascending infection since e.g. hematogenous spread as well has been documented to be the reason for bacterial colonization in other mammals and may contribute to the genital tract microbiota (for review see Baker *et al*. (2018). In general, *Staphylococcus* species have been identified not only as part of the uterine microbiota ( Chen *et al.,* 2017; for review see Baker *et al*., 2018) but also of pancreatic and breast (healthy as well as benign and malignant disease) microbiota ( Thomas *et al*., 2018; Urbaniak *et al.,* 2016). Accordingly, the mere presence of *Staphylococcus* can be supposed not to be associated with bacteremia in most women. Interestingly, in HeLa cells *Staphylococcus epidermidis* isolated from breast cancer patients was found to be able to induce DNA double-stranded breaks ( Urbaniak *et al*., 2016) *.*

## Summary, weaknesses of the hypothesis, and conclusions

We have addressed here the genetically distinct group of UL showing mutations of *MED12*. These mutations apparently act as the drivers in the majority of UL particularly occurring in women having multiple nodules and make UL in general the most frequent symptomatic human tumors at all. Nevertheless, in almost all cases these nodules appear to be of independent origin rather than clonally related. As other types of UL, those carrying *MED12* mutations undergo regression after menopause thus restricting symptomatic UL almost exclusively to women in their reproductive ages. This may point to so far unknown hormonally-regulated factors interacting with the mutated *MED12*. Hormone-dependent growth also characterizes fibroadenomas of the breast, one of the two other tumors frequently displaying the same type of *MED12* mutations, while this is not the case for CLL. However, the unique pattern of *MED12* mutations as well as their high prevalence among frequent human tumors prompted us to speculate about factors that might support the occurrence of this type of genetic alterations in a non-random fashion. What can be noted is a hotspot region within exon 2 of the gene which is affected by most single nucleotid exchanges, deletions, as well as indels.

Deduced from the types and patterns of *MED12* mutations in human tumors we have presented evidence supporting the idea that the driver mutations of *MED12* do not result from selection of random mutational events but rather can be explained by targeted DNA-strand breaks and their repair, respectively. Then, as the second part of our hypothesis, we have advanced the idea that the interaction of nucleic acid sequences of the human microbiome with the common hotspot of *MED12* mutations may constitute the initial event. This involvement of infectious agents would also explain the frequent multiplicity of the corresponding lesions at least in UL. Affected by these drivers, the mutated cells may give rise to clonally independent tumors or, even much more frequently, the mutations may not exert a tumor-driving potential as e.g. in case of frame-shift deletions occurring in no more than single cells thus remaining undetected. In case of “active mutations”, additional factors such as the site of origin, angiogenetic support, and the type of *MED12* mutation, may endow the resulting monoclonal lesions with a different growth potential.

To stimulate further discussion a possible interaction of a sequence of *Staphylococcus aureus* with this hotspot has been considered in more detail as depicted in Figure 7 and Figure 8. As to the initial stage of tumor development, the clearance of R-loops resulting from a hybrid helix between a human target cell and bacterial RNA may simultaneously give rise to multiple mutated cells. 

This hypothesis, though able to explain main biological characteristics of UL and fibroadenomas of the breast, has some weaknesses and leads to further questions. One point relates to the transmission of the pathogenetic bacteria. Do they act after sexual transmission and ascending infection, respectively? Since parity is well-documented leading to a decreased leiomyoma burden, the argument for reproductive tract infections is not supported. Similarly, parity is also associated with increased risk of postpartum iatrogenic infections. On the other hand, the presence of the bacteria and their nucleic acids has not necessarily to be associated with sexual i.e. ascending transmission and clinically manifest pelvic infections. Interestingly, *Staphylococcus* forms part of the normal microbiota of some organs and its circulating nucleic acids may thus derive from bacteria/infections of other sites of the body. As to the transfer of such nucleic acids into the host cell, the internalization of naked bacterial RNA or, alternatively, transfer *via* bacterial extracellular vesicles or target cells that first internalize the bacteria have to be considered. Of note, *Staphylococcus* is a facultative intracellular pathogen ( Sendi & Proctor, 2009) with various types of host cells having the ability to internalize the bacteria ( Rollin *et al*., 2017). Furthermore, it remains to be investigated which mechanisms can lead to an unfolding of short hairpin transcripts and their hybridization to genomic DNA.

In summary, we feel that the exceptional epidemiology of tumors affected by *MED12* mutations as well as the patterns of their mutations warrant unusual explanations helping to decipher the etiology and molecular pathogenesis of some of these highly frequent human tumors. 

## Data availability

All data underlying the results are available as part of the article and no additional source data are required.
