# Supplementary material for: Factors targeting MED12 to drive tumorigenesis?
Source: F1000Res. 2018 Dec 13;7:359. Originally published 2018 Mar 22. [Version 2] doi: 10.12688/f1000research.14227.2 (PMC6325618; doi:10.12688/f1000research.14227.2)
Supplement: Peer review history

# Peer review: Referee response for version 2

**DOI:** 10.5256/f1000research.18776.r41940

**Published:** 2019-01-09

**Authors:** Eric Glasgow

**Affiliations:** 1 Department of Oncology, Georgetown University, Washington, DC, USA

The authors have addressed the major issues concerning the clarity and logic of the argument. The new section, Summary, weaknesses of the hypothesis, and conclusions, is an excellent addition. Most of the grammatical and stylistic corrections have been made, however, since new text has been added there are still a lot of English language problems throughout the paper. A copy editor for English language would be very helpful for the revision?

I have read this submission. I believe that I have an appropriate level of expertise to confirm that it is of an acceptable scientific standard.

# Peer review: Referee response for version 2

**DOI:** 10.5256/f1000research.18776.r41939

**Published:** 2019-01-08

**Authors:** Takeshi Kurita

**Affiliations:** 1 Departmentof Cancer Biology and Genetics, Comprehensive Cancer Center, Ohio State University, Columbus, OH, USA

The revised manuscript should be indexed with minor edits to correct errors. Although the hypothesis that a bacterial transcript causes human neoplasms through a mutagenesis of *MED12 *lacks experimental and observational evidence, it is not a major weakness for an opinion paper. The provocative discovery of a homologous sequence between *Staphylococcus *genome and human *MED12 *gene merits further discussion. 

 In my opinion however, the high incidence of *MED12 *mutation can be explained by random mutations and subsequent selection.  As I have already explained in the critique for the original version, the incidence of tumorigenic *MED12* mutations is expected to be high: diverse *MED12 *mutations appear to be tumorigenic, and monoallelic mutations are dominant, as *MED12 *is a X-linked gene. The symmetric distribution of the beginning and ending bases of deletions around the mutation hotspot in *MED12 *exon 2 (Figure 4) demonstrates that hotspots for missense mutation are also hotspots for deletion mutations. Thus, the distribution pattern is explained by a critical role of the hotspot amino acids in the tumorigenic activity of mutant MED12. Additionally, there is no known mechanism through which a naked nucleic acid of microorganism origin is internalized and hybridized with genomic DNA in human cells.  Therefore, the sequence homology seems to be the only supporting data for the provocative hypothesis. To establish the feasibility of the hypothetical model, the presence of *Staphylococcus *hairpin RNAs must be demonstrated in human tissues.  On the other hand, the absence of *Staphylococcus *infection or *Staphylococcus *hairpin RNAs in tumor tissues does not reject the hypothesis because pathogenic *MED12* mutations should occur years earlier than tumor presentation. 

 The approach by which the authors identified the homologous sequence to the mutation hotspot of human *MED12 *in bacterial genomes does not determine the frequency of homology between such bacterial transcripts and human genes. Nevertheless, if free RNAs of microorganisms can trigger mutagenesis in human cells, there should be genomic loci other than *MED12 *exon 2 that would also be targeted by microbial RNAs through the same mechanism.  Thus, a systematic screening of bacterial genomes for homologous sequences to human genomic loci, in which somatic mutations are frequently detected, may provide evidence for the hypothetical model. 

 Errors

 There is an extra “a”: “In general, epidemiological studies have "a" revealed a lower risk of fibroid development associated with parity which may be related to tissue remodeling during pregnancy.”

 A period is missing between sentences “Between 50% and 60% of the FA harbor *MED12* mutations ( Lim *et al.*, 2014; Mishima *et al.*, 2015; Pfarr *et al.*, 2015)”and “Histologically, the ….”.

I have read this submission. I believe that I have an appropriate level of expertise to confirm that it is of an acceptable scientific standard.

# Peer review: Referee response for version 1

**DOI:** 10.5256/f1000research.15482.r36051

**Published:** 2018-07-31

**Authors:** Jose M. Teixeira

**Affiliations:** 1 Department of Obstetrics, Gynecology and Reproductive Biology, College of Human Medicine, Michigan State University, Grand Rapids, MI, USA

The opinion article by Bullerdiek and Rommel provides an interesting hypothesis that is partly supported by the unusual role of MED12 in the origin of uterine leiomyoma.  MED12 is mutated in these benign tumors at a hotspot location at the 5’ end of exon 2.  How these mutations arise in the myometrium and how the mutant MED12 drives leiomyoma development are not known.

 I agree with many of the previous reviewers’ comments and will limit my comments to a few items so as not to be too repetitious.  For example, I agree that there is no evidence that the MED12 mutations constitute a gain-of-function in the protein. Indeed, if MED12 mutation alone is sufficient for tumorigenesis, it would have been observed in a variety of other tumor types.  In fact, no mechanisms for tumorigenesis unique to leiomyomas have been reported.  Thus, how MED12 mutation leads to leiomyoma development is not known at this point.  This should be changed in the text.

 I also agree that the argument for foreign RNA from bacterial infection causing MED12 mutation, although quite novel and certainly interesting, is weak.  In addition to the reasons already discussed, ascending infections, such as the reproductive tract infections described by the authors, are likely to rarely involve the myometrium. In the case of S. aureus, it is even more unlikely because myometrial infection with that organism is likely associated with bacteremia, which is even more rare and cannot account for the prevalence of MED12 mutant fibroids in women.  Also, although leiomyomas are normally found in reproductive age women, that doesn’t necessarily mean that sexually active women are more prone to the disease because of possible sexually transmission of pathogenic bacteria. Since parity is associated with a lower leiomyoma burden, the argument for reproductive tract infections being the culprit is not supported.  Parity is also associated with increased risk of postpartum iatrogenic infection, but again parity is associated with decreased risk for leiomyomas.  These caveats should be included in the text.

 There is something special about the very common MED12 mutations in that hotspot and high prevalence of uterine leiomyomas (and the not-so-common fibroadenomas) in reproductive age women.  It is possible that a hormonally-regulated factor expressed only in myometrium (and breast stroma) could be interacting with wt MED12 on the site where the hotspot mutations alter the protein structure, and mutation disrupts that interaction.  How those MED12 mutations occur and why the tumors/fibroadenomas develop only in those tissues is a mystery that needs to be resolved in order to develop therapies targeting the mechanisms involved.

I have read this submission. I believe that I have an appropriate level of expertise to confirm that it is of an acceptable scientific standard, however I have significant reservations, as outlined above.

# Peer review: Referee response for version 1

**DOI:** 10.5256/f1000research.15482.r34711

**Published:** 2018-06-19

**Authors:** Eric Glasgow

**Affiliations:** 1 Department of Oncology, Georgetown University, Washington, DC, USA

This opinion article by Bullerdiek *et al. *reviews the types and etiologies of tumors associated with MED12, and put forward a hypothesis regarding the mechanism of *MED12* mutation. MED12-associated tumors are understudied considering their high frequency, probably because they are most commonly associated with benign tumors. However, a better understanding of the etiology of these tumors would likely contribute substantially to our knowledge of the genetic mechanisms of tumorigenesis. The authors discuss the main types of tumors caused by *MED12* mutations: uterine leiomyomas (ULs), fibroadenomas of the breast, and chronic lymphocytic leukemias. They point out that the characteristics of ULs associated with *MED12* mutations differ markedly from those of ULs with other genetic causes. Within the same patient, multiple tumors with *MED12 *mutations of independent clonal origin are common. Bullerdiek *et al. *illustrate the presence of a mutational hotspot within the *MED12 *sequence with homology to tRNAs of multiple human pathogens. These qualities are suggestive of a genome editing mechanism warranting further investigation.

 Important changes:

 -The gene name! The gene name is mediator complex subunit 12 (MED12), not Mediator Subcomplex 12 as stated in the article.

 -In the Abstract it is stated “(MED12)…presumed classification as gain-of-function mutations…”, and in the Introduction “…deletions and, more rarely, indels, usually affecting exon 2 or the intron 1/exon 2 boundary are found which always leave the reading frame intact indicating gain-of-function mutations”. The term gain-of-function should be removed. The function of MED12 is unknown and there is no evidence suggesting that these mutations are gain-of-function.

 Major issues:

 -In Figure 2B, it is unclear how many patients in total were analyzed for tumor counts or whether the multiple *MED12-*mutation positive UL were concentrated in a small number of patients or spread evenly across patients analyzed.

             -Additionally, the labels appear to be switched in the key of Figure 2.

 -A major problem is that the authors claim that the occurrence of multiple ULs can be exclusively attributed to *MED12 *mutation. However, although the authors provide ample quantitative evidence of multiple ULs resulting from *MED12 *mutation within the same patient, the authors show no data regarding the occurrence of multiple ULs with other genetic mechanisms (i.e. *HMGA2 *arrangements). In order to make the claim that multiple UL is a feature unique to *MED12-*mutated tumors, the authors should provide data on non- *MED12*-mutated multiple UL for the sake of comparison. If the number of non- *MED12*-mutated multiple UL is zero, this should be made clear within the text of the article.

 -In reference to Figure 5, it is unclear whether the CLL mutations (yellow) cluster around the mutational hotspot as do the UL and fibroadenoma mutations, although the text of the article claims that the CLL mutations also cluster around the hotspot.

 -The authors point out that the evidence for a connection between infection and UL is dubious thus far, yet they go on to propose a mechanism for the role of infection in UL pathogenesis. To make a convincing argument, the authors must clarify why they still consider a pathogenic mechanism worth considering.

 -The authors should provide more detail about the pathogens with tRNA sequences matching the mutational hotspot. What is known about the involvement of these pathogens in pelvic infections? Does the prevalence of pelvic infections involving these pathogens approximately match the prevalence of UL? Is there any existing evidence for association between these pathogens and UL?

 Minor issues:

 -Second paragraph of the introduction: “As in the benign tumors, however, mutations of that gene occur as apparent driver mutations in a predominant subset of uterine leiomyomas (Mäkinen *et al.*, 2011; Markowski *et al.*, 2012; McGuire *et al.*, 2012), constituting the by far most frequent human symptomatic tumors of all.” Do the authors mean “As in the malignant tumors...” Also, “that gene” is vague. Replace with *MED12.*

 -Second paragraph introduction: “Furthermore, the same type of *MED12 *mutations was found in two canine vaginal leiomyomas (Markowski *et al.*, 2013a)” can be changed to “Furthermore, the same type of *MED12 *mutation was found in two canine vaginal leiomyomas (Markowski *et al.*, 2013a)”

 -Third paragraph introduction: “Predominantly, the mutations are clustered in the 5 ´ region of exon 2 of the gene with only a few mutations affecting the intron 1-exon 2 boundary or, much rarer, exon 1 or the exon 1-intron 1 boundary” can be changed to: “Predominantly, the mutations are clustered in the 5 ´ region of exon 2 of the gene with only a few mutations affecting the intron 1-exon 2 boundary or, more rarely, exon 1 or the exon 1-intron 1 boundary”

 -The heading “Introducing three tumor entities displaying a unique type of *MED12* mutations” should be changed to “Introducing three tumor entities displaying *MED12* mutations”

 -Under subheading: Uterine Leiomyomas – the most frequent symptomatic human tumors, “Depending on their location it can be distinguished between submucosal, intramural, and subserosal UL” can be changed to “Depending on the location, the difference between submucosal, intramural, and subserosal UL can be distinguished”

 -The subheading “Fibroadenomas of the breast - Frequent benign tumors of adolescent and young women” can be changed to “Fibroadenomas of the breast - Frequent benign tumors in adolescent and young women” and the subheading “Chronic lymphocytic leukemias - most frequent leukemia of the adults” can be changed to “Chronic lymphocytic leukemias - most frequent leukemia in adults”

 -Under the heading “A closer look at the molecular pathogenesis of uterine leiomyomas,” the sentence, “According to the high prevalence of uterine leiomyomas *MED12 *mutations are by far best investigated in this tumor type” can be changed to “ *MED12 *mutations are by far best investigated uterine leiomyoma tumor type.”

 -Second paragraph under the subheading: “Leiomyomas with *MED12 *mutation constitute their own genetic subtype which is also characterized by a distinct clinical and histopathological appearance,” the sentence “Accordingly, both mutations allow the two major genetic subtypes of UL to be distinguished, and the question arises whether or not the genetic subtypes are also reflected by a different clinical behavior and histopathology” can be changed to “Accordingly, these mutations allow the two major genetic subtypes of UL to be distinguished, and the question arises whether or not the genetic subtypes are also have different clinical behaviors and histopathologies”

 -The second paragraph under the subheading: “The percentage of *MED12* **-**mutated tumors is positively correlated with the total number of tumors per patient,” the sentence “Along with previous data this distribution confirms that, as for its pathogenesis, the occurrence of multiple leiomyomas near exclusively can be attributed to just one genetic mechanism, i.e. *MED12 *mutations” can be changed to: “Along with previous data this distribution confirms that the occurrence of multiple leiomyomas nearby can be exclusively attributed to just one genetic mechanism, i.e. *MED12 *mutations.”

 -At the end of the first paragraph under the heading: “A closer look at the patterns of *MED12 *mutations seen in various benign and malignant tumors” the word respectively is unnecessary.

 -Under “Hypothesis and Opinion” the sentence “Nevertheless, additional factors favoring this multitude of tumors carrying the same type of mutations despite their independent clonal origin have remained enigmatic” should be changed to “Nevertheless, additional factors favoring this multitude of tumors with independent clonal origin carrying the same type of mutation have remained enigmatic.”

 -Also, “In addition to these both models possibly accounting for other genetic subtypes, as a third alternative factors as in particular infectious agents warrant consideration” should be changed to “In addition to these models, the potential roles of infectious agents warrant consideration”

 -Also, “As to another virus of the Herpes group, a recent study failed to reveal a significant association between *HSV-2* seropositivity and the presence of fibroids (Moore *et al.*, 2016) and in general not convincing evidence for viruses involved in the pathogenesis of UL has been presented” should be changed to “As to another virus of the Herpes group, a recent study failed to reveal a significant association between *HSV-2* seropositivity and the presence of fibroids (Moore *et al.*, 2016) and in general no convincing evidence for involvement of viruses in the pathogenesis of UL has been presented”

 To summarize, the authors build a fairly convincing argument for the need to more closely study the mechanism of *MED12 *mutation in tumorogenesis. However, as detailed above, there are multiple areas in which additional evidence and clarification is needed to support the authors’ claims. Additionally, correction of several grammatical errors and awkward phrasing throughout the manuscript would greatly improve readability.

I have read this submission. I believe that I have an appropriate level of expertise to confirm that it is of an acceptable scientific standard, however I have significant reservations, as outlined above.

# Peer review: Referee response for version 1

**DOI:** 10.5256/f1000research.15482.r33313

**Published:** 2018-04-30

**Authors:** Takeshi Kurita

**Affiliations:** 1 Departmentof Cancer Biology and Genetics, Comprehensive Cancer Center, Ohio State University, Columbus, OH, USA

This Opinion Article proposes a provocative idea that transcripts of *Staphylococcus aureus *contribute to an exceptionally high incidence of *MED1*2 exon 2 mutations among human tumors. 

 After providing an excellent summation of the current literature on demographics and characteristics of *MED12 *mutations in human neoplasms, the authors propose an intriguing concept: the involvement of mutagenic nucleic acids of bacterial origin in human tumors, based on the discovery of a Staphylococcus genome sequence of which the transcript may form an R-loop with human MED12 gene on the mutation hotspot. This novel concept merits further discussion among researchers of diverse biomedical fields. 

 There are several issues that I recommend the authors to address.

 I. The importance of the idea would increase if the following issues are resolved.

- The methods of database search for homologous sequences and the statistical significance of the finding should be described. In other words, it is unclear how frequently such homologous sequences to the human genome appear in the genome of pathogenic microorganisms, and what proportion of such homologous sequences can form an R-loop with human genome if they are transcribed.  
- The palindromic sequence is located at 30 bp from the 3’ end of tRNA-Leu on the reverse strand in *Staphylococcus aureus *genome. Since it is not a typical template for transcription, inclusion of references that suggest transcriptional activity in such genomic regions of bacteria would help further discussion by increasing the feasibility of the proposed model.
- It would also be helpful to include a discussion on the possible mechanisms through which naked transcripts of low-copy number in the circulation could possibly reach the target locus in the genome of myometrial cells. For instance, internalization of shRNA for RNAi is achieved by viral transduction or chemical/physical transfection of shRNA expression vectors. 
- The authors may speculate the mechanisms that unfold the minimum free energy structure of short hairpin transcripts and facilitate the hybridization to the genomic DNA. For example, shRNA is processed into siRNA, and then siRNA forms a complex with cellular proteins to elicit RNAi effect. 

II. There are issues in data presentation

- Regarding the title of Figure 2: “Single and multiple MED12-mutation positive uterine leiomyomas”, the adjectives “single and multiple” could modify “MED12-mutation”.  Adding a hyphen ( *MED12*-mutation-positive) and using of “solitary” instead of “single” would improve the readability.
- Error: The labels for single and multiple UL groups in Figure 2 are switched. 
- The graphs in Figures 2, 3 and 4 are the same data in different presentation format. The Figures 3 and 4 seem to be redundant.

III. There are other factors that also likely contribute to the high prevalence of patients with multiple *MED12 *mutant ULs.

1. A single hit on the active *MED12 *allele is sufficient for the pathogenesis of ULs. Human cancer cells usually carry multiple putative driver mutations even at the earliest stage. In contrast, most *MED12 *mutant ULs do not carry additional mutations, suggesting a *MED12 *mutation is sufficient to drive UL pathogenesis. Since the *MED12 *is on X-chromosome, a single hit on the active allele of *MED12 *has dominant effect. Hence, the development of UL through *MED12 *mutations should occur at a significantly higher rate compared to the neoplasms that require multiple genetic lesions for pathogenesis.
2. Diversity of pathogenic *MED12 *mutations increases the prevalence of *MED12*-mutant ULs.  Generally, driver missense mutations of human neoplasms are very specific. For instance, nearly all adult-type granulosa cell tumors carry *FOXL2 *c.402C>G (C134W) missense mutation. While single base replacements in the hotspot triplet bases can also result in conversion of C134 to F, S, Y, R and G, only C134W is pathogenic. In contrast, a variety of *MED12 *mutations, ≥ 10 missense and > 30 indel mutations, are associated with ULs. For instance, missense mutations that convert MED12 G44 to D, S, V, R, C and A are all pathogenic. Hence, even if the mutation rate per nucleotide is equal throughout the human genome, the incidence of pathogenic *MED12 *mutations should be many times higher than other pathogenic mutations. 
3. UL is a hormone dependent tumor.  Another key factor that this Opinion Article does not address is the systemic hormonal environment. The pathogenesis of UL depends on estrogen and progesterone. Since ULs are counted only when they grow to a grossly recognizable size, patients with endocrine profiles favorable to the growth of ULs should have a higher number of tumors even if the incidence of pathogenic mutations in myometrial cells is equal among all women. 

Discussion of these factors is not essential, as the model proposed in this article would work independently. Nevertheless, these additions would help balance the discussion.

I have read this submission. I believe that I have an appropriate level of expertise to confirm that it is of an acceptable scientific standard, however I have significant reservations, as outlined above.
